# Development of a Sandwich-Type *sxtA4* Electrochemical Biosensor for Proactive Environmental Monitoring of STX-Producing Microalgae

**DOI:** 10.3390/bios16050252

**Published:** 2026-04-30

**Authors:** Hyunjun Park, Seohee Kim, Minyoung Ju, Yunseon Han, Yoseph Seo, Junhong Min, Hyeon-Yeol Cho, Taek Lee

**Affiliations:** 1 Department of Chemical Engineering, Kwangwoon University, 20 Gwangwoon-Ro, Nowon-Gu, Seoul 01897, Republic of Korea; andy9760@kw.ac.kr (H.P.); rlatjgml31@kw.ac.kr (S.K.); minyoung9585@kw.ac.kr (M.J.); hys02100@kw.ac.kr (Y.H.); akdldytpq12@kw.ac.kr (Y.S.); 2 School of Integrative Engineering, Chung-Ang University, 84 Heukseok-Ro, Donjak-Gu, Seoul 06974, Republic of Korea; 3 Department of Integrative Biotechnology, Kookmin University, 77 Jeongneung-Ro, Seongbuk-Gu, Seoul 02707, Republic of Korea

**Keywords:** electrochemical biosensor, saxitoxin, sandwich-type, ACEF technique

## Abstract

Saxitoxin (STX), produced by certain harmful algal bloom (HAB) species, bioaccumulates through the food chain and can cause paralytic toxicity in humans, potentially resulting in fatal outcomes. To date, STX detection has primarily been conducted under laboratory-controlled conditions, and the availability of a gold-standard method for the proactive monitoring and prevention of HAB-induced secondary damage remains limited. Therefore, the present study introduces an electrochemical-based biosensor that is capable of early monitoring of STX in HAB-occurred environments. The conserved region of *sxtA4*, a nucleic acid precursor that is essential for STX biosynthesis, is immobilized on the sensing membrane surface in a sandwich structure. In this process, target detection is recognized as an electrochemical signal by a methylene blue-labeled detection probe, and the reliability of biosensing is supplemented by an electrochemical trend that is opposite to DNA binding. The application of an alternating current electrochemical flow technique achieves more sensitive detection at attomolar levels and rapid measurement within 10 min than a conventional DNA biosensor based on hybridization. In addition, the designed biosensing structure selectively detects STX-synthesizing and non-synthesizing dinoflagellates significantly. The proposed platform can utilize the identification of STX-induced secondary damage of HAB and provide insight into a field-ready biosensor based on its characterization and detection performance.

## 1. Introduction

Harmful algal bloom (HAB) has emerged as a growing global environmental concern, with increasing frequency and escalating ecological and public health impacts. Among their most severe consequences, paralytic shellfish poisoning poses a significant threat by inducing neuromuscular paralysis through the disruption of sodium channel function in muscle cells [1]. This condition is primarily caused by saxitoxin (STX), a potent neurotoxin produced by specific HAB species, and is transmitted through the consumption of contaminated marine organisms. Clinical manifestations of STX exposure typically include mild paralysis and gastrointestinal dysfunction, whereas higher exposure levels can lead to respiratory paralysis and death [2]. STX further exists as a diverse group of structurally related analogs [3], which can be metabolically transformed into more toxic forms within marine organisms [4]. Environmental stressors associated with climate change have been reported to exacerbate STX toxicity in aquatic systems [5]. These dynamic environmental and biochemical factors collectively complicate risk assessment and underscore the need for rapid field-ready detection strategies. The development of robust STX detection platforms is therefore essential for effective environmental monitoring and the prevention of toxin-related marine hazards.

Conventional approaches for STX detection, including mouse bioassays [6], enzyme-linked immunosorbent assays [7], and liquid chromatography [8], have been widely employed for toxicity assessment and risk prediction. However, variability in toxicity among STX analogs complicates the establishment of standardized reference compounds for accurate quantification [9]. In addition, these methods are largely confined to laboratory settings, limiting their applicability for rapid and field-ready detection. Biosensors have emerged as a compelling alternative, offering portability and user-friendly operation that overcome spatial and technical constraints. Their ability to detect target analytes with minimal procedural requirements, without the need for specialized expertise, enhances their suitability for field application [10,11]. Comparable analytical performance to conventional devices, combined with cost efficiency and rapid response, further supports their potential as practical tools for real-time monitoring [12]. Moreover, biosensors can be tailored to specific analytical targets, enabling the construction of versatile detection platforms for a wide range of biomolecules [13]. From the perspective of the central dogma of molecular biology, nucleic acid analysis provides indirect insight into the presence and abundance of downstream biomolecules. The STX biosynthetic gene (*sxt*) is independently encoded in microalgae [14], and its expression levels have been shown to correlate with STX production [15]. Therefore, detection of *sxt* enables the omission of direct STX extraction and provides a potential means to assess the toxic impact of diverse STX analogs.

**Figure 1:**
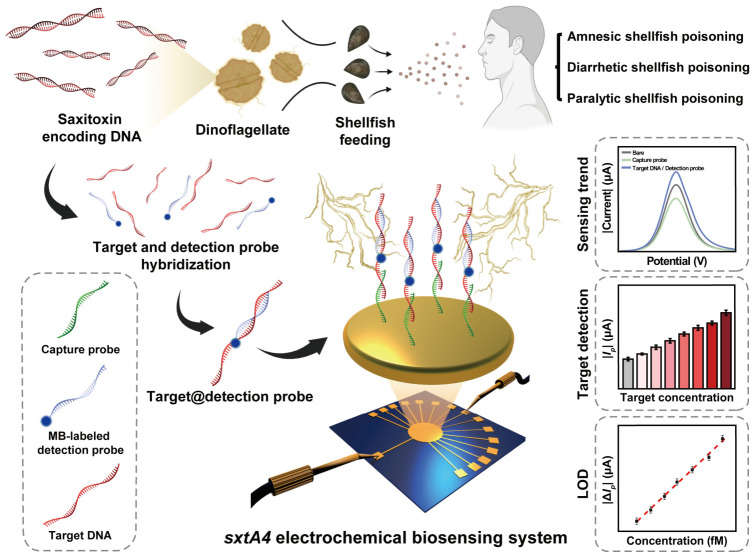
Schematic illustration of fabricated *sxtA4* electrochemical biosensing system. The graphic was created with BioRender.com and used with permission.

This study introduces a *sxtA4* detectable electrochemical biosensor that is capable of enabling proactive monitoring of STX. The *sxtA4*, a conserved biosynthetic precursor involved in STX production in toxic dinoflagellates, serves as a key genetic marker for the selective identification of STX-producing species [16]. The target was functionalized onto the sensing interface through the formation of a sandwich complex with dual probes. Under the biosensing structure construction process, methylene blue (MB) was employed as an electrochemical label for qualitative and quantitative analysis of the target. This strategy exhibits an inverse signal trend, relative to conventional nucleic acid electrochemical sensing, allowing clear discrimination based on target presence. Alternating current electrothermal flow (ACEF) was used to accelerate the target–detection probe (Dp) complex formation and enhance detection sensitivity. ACEF induces periodic electrical potentials to the electrode, generating microscale fluid flow within the drop-casted solution [17]. This effect enhances target-probe binding events and reduces the reaction time to within 10 min compared to natural diffusion, providing attractive insights for field-ready detection platforms [18]. Potential interferences and sensing performance were systematically evaluated under in vitro conditions. The practical applicability of the platform was further examined using biosensing assays with biologically derived genomic DNA (gDNA). Ultimately, the novelty of the proposed *sxtA4* biosensing system is as follows: universal sample detection regardless of target amplification, improved biosensing reliability and sensitivity through the application of MB-labeled Dp, and reduced unamplified target detection time within 10 min by using ACEF technique. The schematic illustration of the *sxtA4* biosensing system is presented in Figure 1.

## 2. Materials and Methods

### 2.1. Materials

The Au round-type micro-gap electrode was manufactured by SNI Technology (Seoul, Republic of Korea). Potassium hexacyanoferrate (II) and potassium hexacyanoferrate (III) were purchased from Sigma-Aldrich (St. Louis, MO, USA). Acetone (ACE; C_3_H_6_O), boric acid (H_3_BO_3_), ethyl alcohol (EtOH; C_2_H_5_OH), 4-(2-Hydroxyethyl)-1-piperazineethanesulfonic acid (HEPES; C_8_H_18_N_2_O_4_S), and tris(hydroxymethyl)aminomethane (Tris; C_4_H_11_NO_3_) were purchased from Daejung (Siheung-si, Republic of Korea). The Ag/AgCl reference electrode was purchased from CH Instruments (CHI111, Austin, TX, USA). *Alexandrium pacificum* (HMIBR-MA39) and *A. pacificum* gDNA (HMIBR-NT386) were obtained from Bank of Bioresources from Island and Coast of Honam National Institute of Biological Resources (Mokpo-si, Republic of Korea). The microalgae for the selectivity test, such as *Microcystis aeruginosa* (FBCC-A59), *Anabaena circinalis* (FBCC-A104), *Leptolyngbya boryana* (FBCC-A310), and *Limnothrix planctonica* (FBCC-A518), were obtained from the Nakdonggang National Institute of Biological Resources (NNIBR, Sangju-si, Republic of Korea). The QIAamp DNA Mini Kit was purchased from Qiagen (Venlo, The Netherlands). The polymerase chain reaction (PCR) clean-up kit was purchased from Bionics (Seoul, Republic of Korea). The agarose powder, DNA size marker, PCR master mix, and 0.5 M ethylenediaminetetraacetic acid (EDTA; C_10_H_16_N_2_O_8_) were purchased from Bioneer (Daejeon, Republic of Korea). The loading star was purchased from Dyne Bio (Seongnam-si, Republic of Korea). The oligonucleotides were synthesized from Bionics, and their sequence details are listed in Table 1.

### 2.2. Biosensing Sample Preparation

The gDNA was extracted using the manufacturer’s manual. PCR was conducted using iCycler (Bio-Rad, Hercules, CA, USA), under the following cycling conditions: pre-denaturation at 95 °C for 5 min, 35 cycles at 95 °C for 30 s, 60 °C for 30 s, 72 °C for 30 s, and final extension at 72 °C for 10 min. The PCR products were confirmed by 1% Tris-borate-EDTA agarose gel electrophoresis at 100 V for 1 h. The DNA was quantified using a multimode reader and converted to molality using Equation (1) [20].

DNA Molarity (M) = DNA Mass concentration (g/L)/DNA length × 660(1)

### 2.3. sxtA4 DNA Biosensor Fabrication

A round-type micro-gap electrode was sonicated using ACE for 10 min. The electrode was then washed using EtOH and DIW and dried with N_2_ gas, respectively. This electrode preparation process was performed after each electrode surface modification step. Then, 10 µL of capture probe (Cp) was dropcast onto the electrode to establish a preliminary biosensing platform. The capture probe and target–Dp complex were functionalized using the ACEF technique, and an input voltage of 3 V and a frequency of 1 MHz were applied for 10 min by a function generator (Tektronix, Beaverton, OR, USA). The sequential biosensing structures’ construction was verified by atomic force microscope (AFM; XE7, Park Systems, Suwon, Republic of Korea) measurements, using a PPP-NCHR microscope probe (Park Systems). AFM analysis was operated in non-contact mode to minimize the damage to biological samples [21], and the surface analysis parameters were self-optimized to improve the analysis resolution.

### 2.4. Electrochemical Measurement Condition

The electrochemical biosensor was constructed as a three-electrode system, and the measurement signal was converted to a workstation (CHI 760E; CH Instruments). The electrochemical reaction was measured using 10 mM HEPES buffer (pH 7.04), containing 5 mM [Fe(CN)_6_]^3−^/^4−^ and 1 M KCL, and represented as Equation (2).

(2) $${\left[\mathrm{Fe}(\mathrm{CN}{)}_{6}\right]}^{4-}\;↔{\left[\mathrm{Fe}(\mathrm{CN}{)}_{6}\right]}^{3-}+e^{-}$$

The working electrode and counter electrode were connected using a probe station. Open circuit potential (OCP) was measured in the range of −1 to 1 V for 400 s, and the data we collected at 0.1 s intervals. Cyclic voltammetry (CV) was performed in the range of −0.10 to 0.60 V at a 0.05 V s^−1^ scan rate in the 2nd cycle. Under the same conditions, square wave voltammetry (SWV) was conducted at a frequency of 15 Hz, an amplitude of 25 mV, and a step height of 4 mV. The electrochemical impedance spectroscopy (EIS) measurements were based on an ideal Randles equivalent circuit [22]. EIS was performed at an applied potential of 0.24 V over a frequency range of 1 to 100,000 Hz with a 5 mV root mean square amplitude.

### 2.5. Statistical Analysis

Electrochemical measurements were repeated at five electrodes (n = 5), each containing 15 working pads, to obtain results for the statistical analysis of reproducibility. The error bars used in each analysis represent the sample standard deviation (s) and were calculated using Equation (3).

(3) $$s=\sqrt{\frac{\sum_{i=1}^{n}{\left(x_{i}-\bar{x}\right)}^{2}}{n-1}}$$

Here, $x_{i}$, $\bar{x}$, and n represent the individual measurements, average value, and number of repeated measurements, respectively. Statistical analysis was performed using SPSS software (Version 19.0; IBM Corp., Armonk, NY, USA) to calculate the standard and equilibration errors for a selectivity test. Significant differences were assessed by one-way analysis of variance (ANOVA) with equal variances, using the Tukey and LSD algorithms for cross-validation. Probability (*p*) values were marked with *** *p* < 0.001, indicating significant differences.

## 3. Results and Discussion

### 3.1. Electrochemical Characterization of the Sensing Membrane

**Figure 2:**
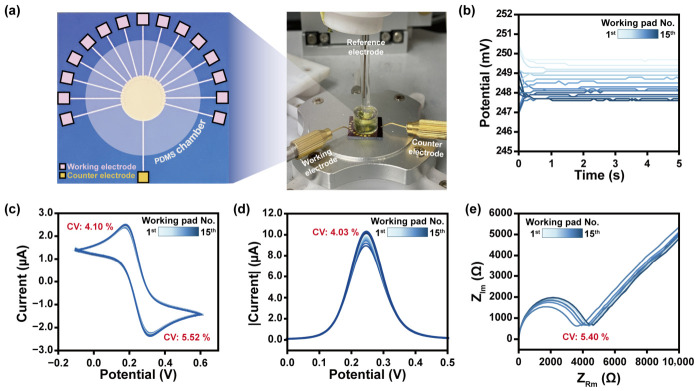
(**a**) Schematic illustration of used electrode and real photo of designed electrochemical biosensing platform. (**b**) OCP results for each working pad. Electrochemical measurements under optimized applied voltage conditions through (**c**) CV, (**d**) SWV, and (**e**) EIS.

The round-type microgap electrode is designed with an identical configuration extending from the counter pad to the working pads, enabling uniform electrochemical measurements [23]. The sensing interface, based on a three-electrode system, comprised 15 individual working pads, allowing for statistically reliable analysis by using one electrode (Figure 2a). This system is capable of sufficiently responding to electrode disconnections and minor defects during field measurements, offering strength in the increased operator convenience and providing reliable, reproducible result analysis. Functionalization of the electrochemical sensing membrane was achieved via drop-casting of a 10 μL sample, providing a practical advantage for field applications where a minimal sample volume is required. The OCP of the electrode stabilized at 0.25 V (Figure 2b), and the subsequent electrochemical measurements were conducted over an applied potential range of −0.1 to 0.6 V. The uniformity of biosensing performance across individual working pads was validated using CV, SWV, and EIS, yielding the following results: CV exhibited an oxidation current of 2.98 μA and a reduction current of −2.90 μA (Figure 2c); SWV showed a peak current (*I_p_*) of 9.80 μA (Figure 2d); and EIS analysis revealed a charge transfer resistance (*R_ct_*) of 3964.23 Ω (Figure 2e). The coefficients of variation for the oxidation current, reduction current, *I_p_*, and *R_ct_* were determined to be 4.10%, 5.52%, 4.03%, and 5.40%, respectively, confirming that the established electrochemical conditions enable highly reproducible and uniform biosensing performance.

### 3.2. Fabrication of sxtA4 Biosensing Platform

**Figure 3:**
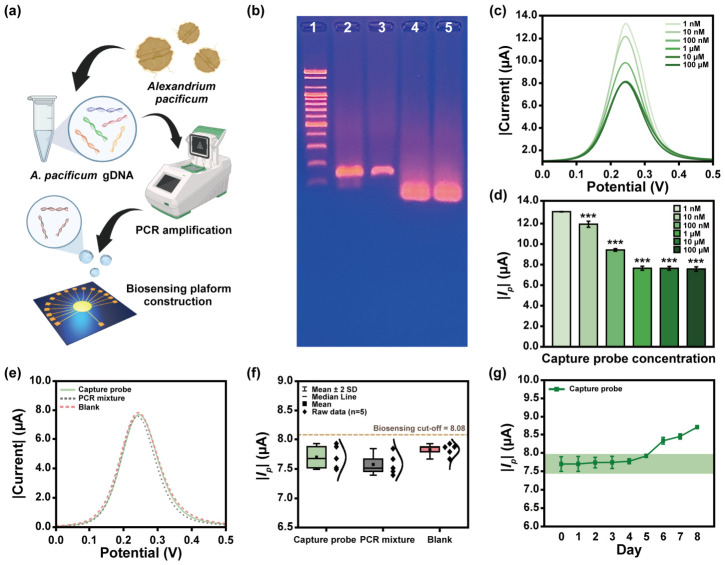
(**a**) Schematic illustration of sample preparation for biosensing performance evaluation. (**b**) *sxtA4* target region amplification results. Each lane’s details are marked as follows: Lane 1: 100 bp DNA ladder; Lane 2: *sxtA4* PCR product; Lane 3: purified *sxtA4* PCR product; Lane 4: *sxtA4* forward primer; and Lane 5: *sxtA4* reverse primer. (**c**) SWV graph and (**d**) |*I_p_*| value at various concentrations of Cp. (**e**) Background noise signals induced in PCR mixtures and blank conditions, and the (**f**) resulting biosensing cut-off calculation. (**g**) Stability test of Cp immobilized sensing membrane. The green box represents the 95% confidence interval based on the initial measurement signal. Significant electrochemical signal differences according to the Cp concentration were determined by ANOVA and were highlighted as *** *p* < 0.001. The graphic of (**a**) was created with BioRender.com and used with permission.

*A. pacificum* gDNA, a representative STX-producing harmful dinoflagellate, was prepared to establish the biosensing system (Figure 3a). A 125 bp target region of the *sxtA4* gene was subsequently amplified via PCR and remained structurally intact without denaturation after purification (Figure 3b). Recent advances in nucleic acid biosensing have increasingly focused on direct detection from extracted samples without amplification [24]. In this context, PCR amplicons were employed exclusively for the validation of sensor fabrication and performance evaluation. The concentration of Cp drop-casted onto the electrode surface exhibited a negative correlation with the measured current (Figure 3c). This phenomenon arises from the highly negative charge of the DNA phosphate backbone, which suppresses electrochemical activity [25,26]. The absolute peak current (|*I_p_*|) decreased progressively up to a Cp concentration of 1 μM, whereas further increases in concentration resulted in negligible current variation (*p* < 0.001) (Figure 3d). Optimization of the bioprobe functionalization is a critical determinant of biosensing performance during sensor construction. Therefore, the electrochemical response observed at 1 μM Cp was identified as the saturation point of surface functionalization and selected as the optimal condition for subsequent biosensing architecture development.

The complex biological matrix generates noise signals through non-specific adsorption on the sensing surface of biosensors [27]. Blocking strategies were introduced to mitigate such interference during biosensor fabrication [28,29]; however, blocking agents alter the physicochemical properties of the sensing interface, which can compromise the detection sensitivity [30,31]. Therefore, the proposed biosensor prioritizes the detection sensitivity and excludes blocking steps while evaluating potential noise sources that may arise during the detection process. The electrochemical signal generated from target-free PCR products differed from the blank by approximately 4.08%, which falls within the experimental error range (Figure 3e). Based on this result, the cut-off value was calculated using Equation (4) [32].

Cut-off value = *I_p_* difference × 3 (4)

The blank exhibited an |*I_p_*| of 7.82 μA and showed the maximum electrochemical signal difference relative to the sensing surface state induced by Cp (Figure 3f). At this point, the *I_p_* difference was 0.11 μA, and the cut-off value for biosensing performance evaluation was set at an |*I_p_*| of 8.08 μA. The electrode functionalized with the Cp maintained output signals within an error range of ±5% compared to the freshly prepared electrode for up to 5 d (Figure 3g), confirming stable and reliable biosensing operation.

**Figure 4:**
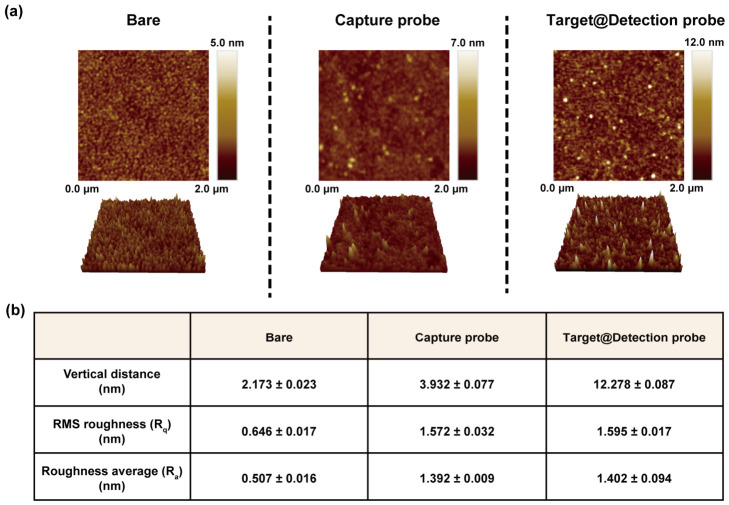
(**a**) AFM analysis and (**b**) surface characterization results according to each electrode functionalization steps.

The sequential construction of biosensing structures changed the characteristics of the electrode surface (Figure 4a). In the surface characteristic parameters, a 2 nm vertical distance (VD) offset of the bare showed that the electrode was suitably prepared for the next modification operation (Figure 4b). The VD measured under Cp and target–Dp functionalized sensing membrane differed from the estimated DNA length [33]. This is due to surface adsorption resulting from measurements in a dry condition and random folding of the DNA. Nevertheless, at each DNA functionalization step, the surface roughness parameters were maintained within the error range, and the VD was gradually increased, indicating the successful sandwich-type biosensing structure construction.

### 3.3. Optimized the Target Detection Condition

**Figure 5:**
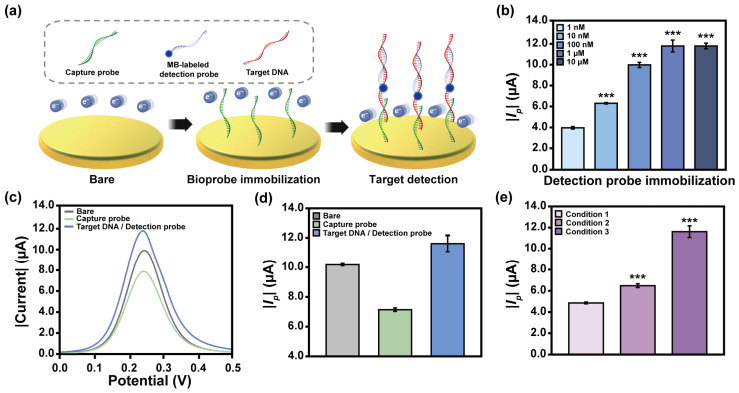
(**a**) Schematic illustration of changes in electrochemical reaction at sequential sandwich-type biosensing structure construction. (**b**) |*I_p_*| value at various concentrations of Dp. (**c**) SWV graph and (**d**) |*I_p_*| values at each electrode modification step. (**e**) Evaluation of target detection sensitivity based on hybridization process. The hybridization conditions are as follows: Condition 1—ACEF applied without pretreatment; Condition 2—target DNA heated at 95 °C, rapidly cooled to 4 °C, followed by mixing with the Dp and immobilized via ACEF; and Condition 3—target and Dp mixture heated at 95 °C, incubated at 55 °C, cooled to 4 °C, and subsequently immobilized via ACEF. Significant electrochemical signal differences according to the Dp concentration and hybridization conditions were determined by ANOVA and were highlighted as *** *p* < 0.001. The graphic of (**a**) was created with BioRender.com and used with permission.

The target *sxtA4* hybridized with Cp and the MB-labeled Dp, forming a sandwich-type biosensing structure. At each stage, the electrochemical signal reflected changes in electron transfer between the sensing membrane and the electrolyte, governed by the sensing membrane functionalization (Figure 5a). Under treatment of 1 μM of the target, the electrochemical signal reached saturation upon addition of 1 μM Dp (*p* < 0.001) (Figure 5b). This optimization of the Dp concentration is critical for achieving sensitive target detection within the Cp–target–Dp hybridized structure. Biosensors based on DNA hybridization inherently depend on the physicochemical properties of nucleic acids [34]. Such signal responses, although consistent, may incorporate noise arising from nonspecific adsorption. Therefore, the introduction of electroactive labels that are capable of modulating and amplifying electrochemical responses represents a key strategy to enhance the detection reliability. The SWV signal, initially suppressed by Cp immobilization, increased significantly upon formation of the sandwich structure with target-bound MB-labeled Dp, exceeding that of the bare condition (Figure 5c). MB facilitates redox reactions by serving as an electron mediator, thereby improving the electrochemical signal output [35]. Under the applied voltage, it is electrochemically reduced to leucomethylene blue (LMB) in a ferri/ferrocyanide solution [36]. LMB transfers electrons to ferricyanide, reducing it to ferrocyanide, and then oxidizing it back to MB. The generated ferrocyanide is then oxidized at the electrode surface, generating the electrochemical current signal. The regenerated MB re-participates in the electrochemical reduction process and catalyzes the redox reaction within the ferri/ferrocyanide solution [37]. The formal potential of MB was −0.356 to −0.056 V [38,39]. The SWV peak was measured at approximately 0.24 V (Figure 5c), which is similar to the formal potential of the ferri/ferrocyanide redox couple (0.22 V) [40]. This result indicated that the signal enhancement was interpreted not by a simple electron shuttle mechanism derived from MB, but by MB-mediated electrocatalytic activation. MB serves as an electrocatalytic mediator that facilitates the electron transfer between the electrode and ferricyanide, while the measured current is predominantly governed by the oxidation of ferrocyanide, corresponding to the final step of the catalytic cycle. Therefore, this contrasts with conventional DNA-induced signal trends, ultimately enabling reliable target detection (Figure 5d).

DNA displacement and hybridization can proceed via isothermal reaction [41]. Thermal-induced denaturation is not strictly required; such treatment enhances the binding interactions between the target and probe [42]. The interaction between the target and Dp was evaluated based on the with and without applied thermal reaction and ACEF (Figure 5e). Denaturing the target DNA to the single-stranded (ss) form reduced the required-hybridization energy of Cp and Dp, resulting in significantly improved detection sensitivity (*p* < 0.001). Sequential denaturation, annealing, and stabilization of the target-Dp complex promoted formation while exposing the 5′ terminus of the target as ssDNA, thereby providing an accessible binding site for Cp. Under this condition, the biosensing signal exhibited the highest sensitivity (*p* < 0.001), demonstrating that a thermal pretreatment of Dp-containing DNA mixture significantly contributes to enhanced target detection performance.

### 3.4. sxtA4 Biosensing Performance Evaluation

**Figure 6:**
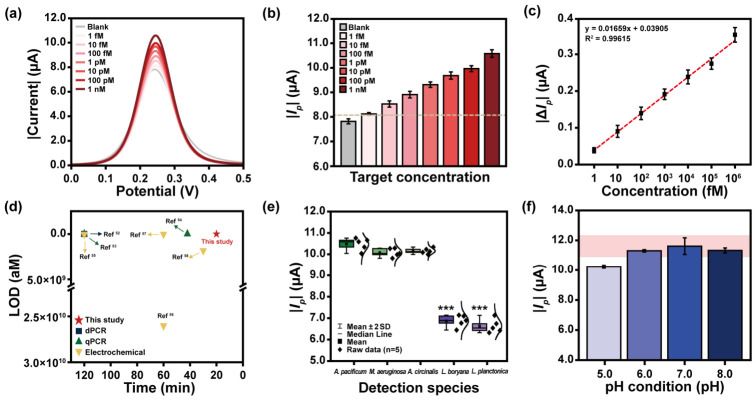
(**a**) SWV graph and (**b**) |*I_p_*| value at various *sxtA4* concentrations. (**c**) Linear regression curves for various concentrations of *sxtA4*. (**d**) Comparison of sensing performance and detection time, including hybridization reaction, between the fabricated biosensor and previous *sxtA4* detection techniques. (**e**) Selectivity test results using microalgae gDNA of 1 nM that produced and did not produce STX. (**f**) Biosensing results according to pH conditions of the measurement environment. The red box represents the 95% confidence interval based on the target detection signal at pH 7.0 condition. Significant electrochemical signal differences between the target species and STX non-producing species were determined by ANOVA and were highlighted as *** *p* < 0.001.

The *sxtA4* detection performance was evaluated using unpurified PCR mixtures, as residual components in the PCR matrix were confirmed to introduce negligible noise, thereby minimizing target loss during sample preparation. An increase in the target concentration exhibited a positive correlation with the formation of the target–Dp complex, resulting in a corresponding increase in the current response (Figure 6a). The |*I_p_*| faithfully reflected the electrochemical response as a function of target concentration (Figure 6b). The lowest target concentration of 1 fM produced a signal above the in vitro biosensing cut-off threshold, ensuring reliable detection. Based on these results, Δ*I_p_* was calculated according to Equation (5).

Δ*I_p_* = |(*I_p_*_,_*_target_* − *I_p_*_,_*_blank_*)/*I_p_*_,_*_blank_*| (5)

Here, *I_p_*_,_*_target_* and *I_p_*_,*blank*_ represent the signals measured in the presence of the target and in the target-free condition, respectively. The biosensing response showed a faithful dependence on target concentration, which was described by a linear regression trend (Figure 6c). The limit of detection (LOD) is a key parameter for evaluating biosensor performance. The universal gold standard is absent for LOD determination, and the reported performance can vary depending on the calculation method employed. To assess the consistency of the fabricated biosensor, the LOD was calculated using equations based on the mean and standard deviation of the blank and the slope of the calibration curve (Table 2).

Here, x, σ, and S represent the average of the blank signal, standard deviation of the blank signal, and slope of the quantification curve, respectively. The sensor exhibited an attomolar-level detection limit for *sxtA4* and demonstrated comparable performance to previous DNA detection techniques, including digital PCR (dPCR), quantitative PCR (qPCR), and electrochemical biosensors (Figure 6d and Table 3). In addition, it demonstrated selective biosensing operation through a significant difference in detection signals depending on the presence or absence of the *sxtA4* (Figure 6e). This represents that the proposed device can achieve universal target detection without interference across different STX-produced species, assessing the potential risks of STX. Various factors present in the measurement environment can affect DNA hybridization and degrade biosensing performance [47,48,49]. Among them, the pH condition plays an important role in changing the toxicity of STX-producing organisms [5]. The *sxtA4* detection was confirmed within a 95% confidence interval in the pH range from 6.0 to 8.0 (Figure 6f), demonstrating the stable hybridization of the sandwich-type biosensing structure. This indicated that the fabricated biosensor could meet robust operating requirements in a typical aquatic environment, industrial wastewater [50], and an acidified environment due to climate change [51].

To summarize, the proposed *sxtA4* biosensor verified the capability for highly selective and sensitive detection of trace-level target genes that are present in environmental samples. The development of a detection technique that is capable of early prediction of HAB occurrence in aquatic environments, along with subsequent secondary impacts, is essential for preventing environmental, economic, and industrial damage. Recent advances in DNA extraction technologies have enabled simplified and rapid systems that are readily applicable in field settings [39]. An integrated platform encompassing target pretreatment and DNA-based biosensing systems is expected to provide clear advantages for environmental monitoring. At the same time, the establishment of a DNA-based biosensing system for STX requires comprehensive screening of the interference factors present in HAB-affected environments, along with a direct evaluation of biosensing performance using environmental DNA samples collected from such conditions.

## 4. Conclusions

Climate change, driven by intensified industrial and economic activities, has led to a continuous increase in the frequency of HAB worldwide. Although various monitoring strategies have been proposed, systems that are capable of enabling early prevention and mitigating secondary damage remain lacking. In the present study, an electrochemical biosensor was developed for proactively detecting the presence of STX at the genetic level. The *sxtA4* was assembled into a sandwich-structured complex incorporating a MB label and subsequently functionalized onto the sensing interface. This design induced an electrochemical response opposite to that typically observed in DNA recognition systems, thereby enabling reliable signal discrimination, depending on target presence, and confirming successful construction of the biosensing structure. The fabricated platform demonstrated attomolar-level sensitivity under in vitro conditions and exhibited negligible noise when using PCR-amplification backgrounds. Furthermore, it represents selective detection that is capable of neglecting gDNA interference in STX biosynthetic microalgae, depending on the presence or absence of the target, and stable operation against environmental variations. The STX contents in STX-producing microalgae varies depending on various environmental factors [59,60]. The copy number of the *sxtA4* shows a correlation with STX toxin content [61]. This is clearly associated with STX production, as relatively low expression levels are observed in non-STX-producing species [62,63]. Under salinity stress conditions, the relationship between the regulation of *sxtA4* and STX accumulation remains insufficiently understood [64], whereas a correlation has been observed between the *sxtA4* transcriptional level and STX equivalents in response to temperature variations [15]. This suggests that the *sxtA4* expression level may reflect environmental STX levels; nevertheless, achieving proactive field monitoring using the developed *sxtA4* biosensor requires a clearer elucidation of the complex molecular mechanisms underlying STX biosynthesis. While overcoming these limitations, the proposed system is expected to provide practical value for field-ready monitoring through the integration of on-site DNA extraction techniques.

## Figures and Tables

**Table 1:** DNA sequence details used in this study.

Name	Remark	Sequence (5′-3′)	Reference
*sxtA4* forward primer	gDNA PCR	CTG AGC AAG GCG TTC AAT TC	[19]
*sxtA4* reverse primer	gDNA PCR	TAC AGA TCG GCC CTG TGA AC	
*sxtA4* Cp	Biosensing	GAA TTG AAC GCC TTG CTC AG-NH_2_	In this study
*sxtA4* Dp	Biosensing	TAC AGA TMG GCC CTG TGA RC-MB	

**Table 2:** Biosensing LOD according to the calculating equations.

Calculation Formula	LOD	Reference
x + 3.3σ	386.02 aM	[43]
x + 3σ	291.44 aM	[44]
3.3σ/S	330.21 aM	[45]
3σ/S	223.13 aM	[46]

**Table 3:** Comparison of the proposed *sxtA4* biosensor and existing detection technique targeting *Alexandrium* sp.

Detection Technique	LOD (aM)	Detection Time (min)	Target	Reference
dPCR	3.3	120	*A.* *pacificum sxtA4*	[52]
qPCR	33	120	*A.* *catenella sxtA4*	[53]
qPCR	118	42	*A. catenella sxtA4*	[54]
Electrochemical	2.478 × 10^7^	120	*A. minutum* DNA	[55]
Electrochemical	2.6 × 10^10^	60	*A. ostenfeldii* rRNA	[56]
Electrochemical	6.91 × 10^4^	60	*A. minutum* DNA	[57]
Electrochemical	1.8 × 10^9^	30	*A. minutum* DNA	[58]
Electrochemical	307.7	20	*A.* *pacificum sxtA4*	This study

## Data Availability

Dataset available upon request from the authors.
